# Welsh Onion (*Allium fistulosum* L.) Seed Physiology, Breeding, Production and Trade

**DOI:** 10.3390/plants11030343

**Published:** 2022-01-27

**Authors:** Gregorio Padula, Xianzong Xia, Roman Hołubowicz

**Affiliations:** Department of Phytopathology, Seed Science and Technology, Poznań University of Life Sciences, ul. Dąbrowskiego 159, 60-594 Poznan, Poland; xianzongx@foxmail.com (X.X.); roman.holubowicz@up.poznan.pl (R.H.)

**Keywords:** Welsh onion, seed storage, breeding, cultivar selection

## Abstract

In the coming years, it is expected that the European Union countries will increase their interest in the use of the Welsh onion (*Allium fistulosum* L.). It is a little-known vegetable from the Far East. Consequently, growers might be asking their extension advisors increasingly about its seed physiology and commercial production. This article provides basic information on the origin of the species, its botanical characteristics, the main breeding directions and achievements, seed production methods and areas, main plant breeding and seed production companies, seed dormancy and the best methods for seed storage, use of the crop and market responses to promotion activities.

## 1. Introduction

The Welsh onion (*Allium fistulosum* L.) is also called stone leek, Chinese onion, Chinese spring onion and Japanese bunching onion. It has been much less known in the world than the traditional onion, *A. cepa* [1]. It has been domesticated in the neighbourhood of Lake Baikal in Siberia near the Altai Mountains in Russia and in Western or North-Western China [2,3,4,5,6,7]. There are still some ancestral species of Welsh onion, e.g., *A. altaicum*. The crop is still popular in Russia, especially in its central part and in Siberia [8,9]. From Siberia, the crop has spread to Japan during the 8th century AD [10]. The first written information about the crop comes from the first century BC [11]. Later on, in medieval times, the crop was brought to Europe from Russia [12]. Based on different botanical classification criteria, the Welsh onion includes five botanical varieties and three cultivar groups. The latter include Chinese, Japanese and Russian cultivars. They all differ in morphological and useful traits, such as number, size and colour of leaves, taste, earliness and overwintering potential.

As early as 30–40 years ago, the Welsh onion (2*n* = 2*x* = 16, genome FF) was grown in the world mostly as an amateur vegetable, except the Far East countries (e.g., Japan, China and South Korea) where it had been produced commercially for many years. It is a perennial to be grown for its early leaves [1,13]. At that time, it was popular to keep the crop in the garden for a period of 3–5 years and then to propagate it through its vegetative parts [1]. The traditional breeding directions of the newly developed Welsh onion cultivars were at that time different from the present ones. In the past, all newly bred Welsh onion cultivars had to have frost resistance to be able to survive winter and thus, were cultivated for some years (as a perennial crop), but they also had to possess high vigour and drought resistance. The overwintering trait affected the fresh leaves productivity; it was a common practice for the growers to increase yield and improve leaf quality by removing bolting inflorescences during the spring season [1]. Moreover, all the cultivars available on the market in the 1980s were open-pollinated (OP), and considering that the Welsh onion is a typical allogamous crop that arose through protandry, with a high outcrossing rate, old cultivars were characterized by a high level of heterogeneity.

## 2. Botanical Characteristics

The Welsh onion is a biennial or perennial herb species of the genus *Allium* and the *Liliaceae* family. Its root system is string-shaped with few and short lateral roots. The stem is short and has a globose or oblate shape. It is surrounded by the base of the leaf sheath, and densely rooted in the lower part [14]. The young leaves are hidden in the leaf sheath and form a round rod-shaped pseudostem with the multilayer sheaths. The underground pseudostem is white whereas the above-soil part is yellow green [4,15]. The leaves are long, cylindrical, hollow, green or dark green, with a smooth and waxy surface. Each plant has 5–8 leaves arranged in a fan shape. Its umbels in the developmental stage are hidden in the membranous involucre. The flowers are small and white, bisexual, and insect-pollinated. The consequence of the latter aspect is the requirement to maintain a “botanical” isolation of the individual cultivars, accessions, breeding lines, etc., to avoid unwanted outcrossing during seed production in order to preserve genetic purity of the crop or breeding line. The flowers are located on an umbel of the head shape. The flowering starts from the top of the head and follows towards the bottom [3,16]. The fruit, called a capsule, consists of three parts, each with two seeds. The mature fruit opens easily. The optimal moment for seed harvest is when 5–10% capsules are open, and seeds are black and hard. The seeds are shield-shaped, with irregular dense wrinkles. In a typical production year, Welsh onion seeds are about half the size of an *A. cepa* seed [16,17]. The 1000-seed weight is 2.4–3.4 g [14].

## 3. Practical Classification

**Figure 1:**
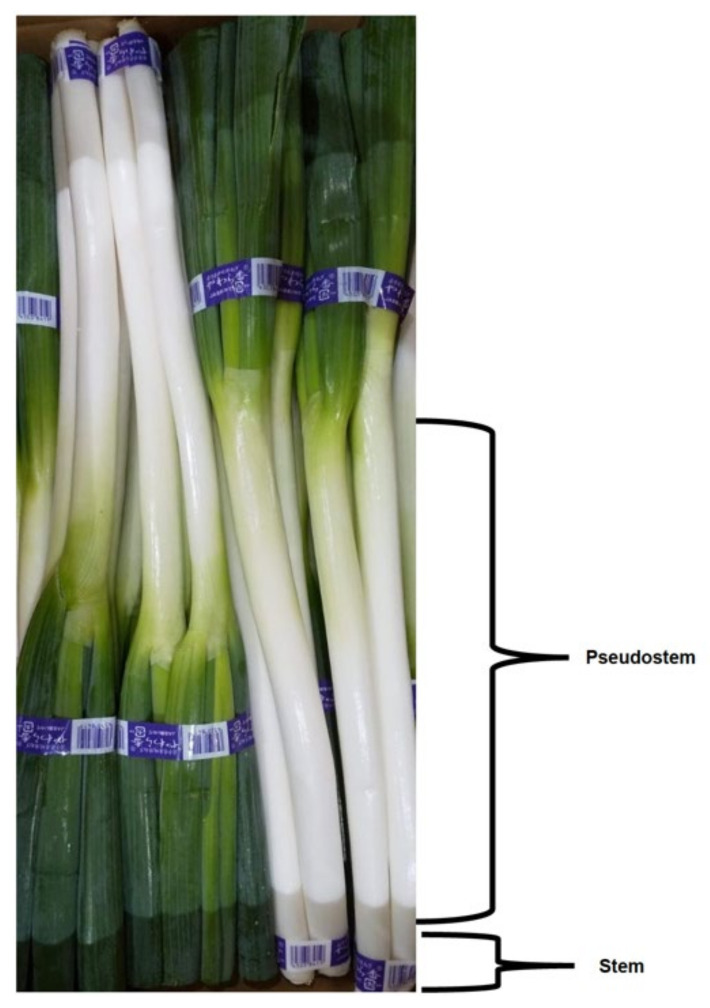
Long white type Welsh onion (Photo G. Padula).

**Figure 2:**
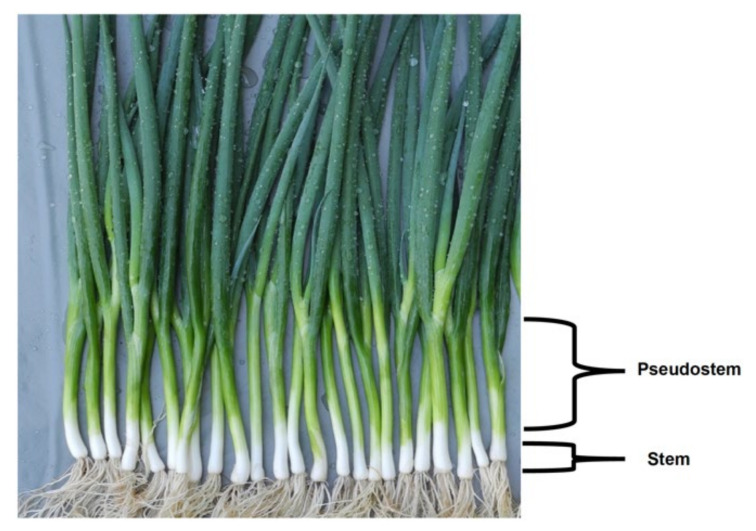
Short white type Welsh onion (Photo G. Padula).

**Figure 3:**
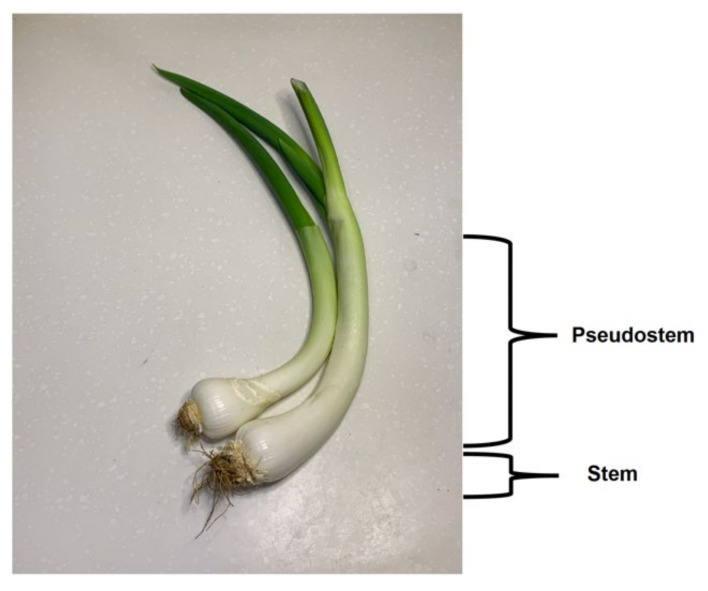
Drumstick type Welsh onion (Photo X.Z. Xia).

For practical crop production, based on the shape of the plant, the Welsh onion can be divided into two main types: common type (also called rod-shaped) with narrow base, and the tillering one with a wide base. However, in Asia, it is more common to divide Welsh onion cultivars into three types based on their pseudostem length [4,14]:

(1) Long white type (Figure 1)—white part over 40 cm. The pseudostem length-to-thickness ratio is bigger than 10. This type has a high yield, light pungency and sweet taste, a high water content, but a short shelf life. Representative cultivars of this type include “*Zhangqiu*”, “*Chishui*” and “*Beijing Gaojiaobai*”.
(2) Short white type (Figure 2)—recognized by a thick and short pseudostem and with a length-to-thickness ratio smaller than 10. The cultivars of this type are resistant to wind lodging and do not require deep soils for cultivation. They give a high pseudostem yield and can be stored on the shelf longer than the plants of the first type. Representative cultivars of this type include “*Shouguang Bayeqi*” and “*Tonghua Xiaohong*”.
(3) Drumstick type (Figure 3)—the pseudostem length is similar to that of the short white type, but the base of the pseudostem is significantly enlarged (wider) and the upper part is obviously thinner. The fully developed pseudostem is of the inverted drumstick or garlic shape. The pseudostem yield is lower, and the flavour is stronger than the other two types. Moreover, plants from this type have shown a long shelf life. Representative cultivars of this type include “*Longyao*” and “*Yinchuan Datou*”.

## 4. Seeds Physiology

As the Welsh onion propagates through seeds, its biology and seed production are important for gardening practice. It has been known already for many years that effective seed storage and longevity are primarily governed genetically, but storage conditions are also important. The storage conditions vary for different species and genotypes/cultivars [18,19,20,21,22]. Amongst many vegetable seeds, Welsh onion seeds belong to the category that do not store long if not properly managed. They lose their germination capacity and vigour when the seeds are kept at room temperature for more than 12 months [5,23]. Therefore, how to extend the storage life of Welsh onion seeds, and thus maintain their germination capacity and vigour during the storage period, is an important problem that needs to be solved in order to preserve its germplasm resources safety [23,24,25,26,27]. Welsh onion seeds, like seeds of other onion-like species, e.g., onion, leek and chive, do not go through a dormancy period after their harvest. It is an important seed physiological character as, if there is high air humidity or rain during the growing period in the field, the seeds may prematurely germinate (sprout) on the umbel of the seed stalk. As all other crops from the onion-like species, they exhibit an orthodox storage behaviour, i.e., they store best at low seed moisture contents, low air temperatures and, if stored in open containers, at low relative humidity of the air [28]. If such conditions are kept, they preserve viability for 6–12 months [20,29,30,31]. A new finding in 2020 showed that a high oxygen partial pressure, instead of high air temperature and relative humidity, also caused the loss of seed vigour of onion-like species seeds after their storage [32].

In an experiment, run on fast-ageing Welsh onion seeds (6% seed moisture content) [33], it was shown that storage in glass vessels for three years at +4 °C, −6 °C and −18 °C, regardless of the used gas atmosphere, did not affect germination, mitotic activity or the frequency of chromosome aberrations and lags in the apical meristem of seedling roots in comparison to the control (kept at room temperature, ≈+20 °C, air). The best conditions for Welsh onion seed storage for the respective temperatures were: +4 °C (argon, carbon dioxide), −6 °C (air, argon, nitrogen, carbon dioxide) and −18 °C (air) as proven by the lowest frequency micronuclei in the root cells of seedlings [33]. Storage of Welsh onion seeds for six years at reduced temperatures (between +4 °C and +9 °C) preserved the germination capacity of the seeds and prevented the occurrence of chromosomal instability in the root meristem cells of the seedlings [34]. A large number of experiments showed that ultradry seed moisture content (below 5%) treatment had a positive effect on the storage duration of Welsh onion seeds [23,25,26,27,35]. Zhang’s team, in 2001, showed that the Welsh onion seeds in ultradry moisture content (2.8%) could be stored at +20 °C for 11 years, while their germination rate, seedling length and enzyme activity were well maintained or remained normal [23]. Li and Wang’s experiment showed that reducing the moisture content of Welsh onion seeds from 8.4% to 5.5%, 5.0%, 3.5%, 3.0% and 2.5%, respectively, (stored in aluminium foil bag at room temperature) did not have a negative effect on their vigour. Furthermore, the processed seeds in Li and Wang’s experiment still maintained a high vigour after artificial ageing treatment (at +50 °C with air humidity around 100%) [25]. Li and Wang further proved, in a continuous experiment, that after 18 months of room temperature storage, the vigour of the seeds in ultralow moisture content (3.5%) did not decrease significantly [26]. It was further reported that the seed moisture content should not be reduced below 2.0% during storage, as such moisture content would cause a decrease of seed vigour [25]. Moreover, the overdry treatment (reduced seed moisture content below 2.0%) would also reduce the seeds’ storage durability and affect genetic integrity [35]. The seeds of different Welsh onion cultivars were stored under ultradry condition for 1 year, in Wang’s experiment, in 2011. The results showed that although the ultradry treatment could be widely used for the seeds of many cultivars of Welsh onion, different cultivars had different response to the ultradry treatment [27]. In another experiment, the harvested seeds had a lower germination when lead and copper were in the soil in which the plants had grown [36].

## 5. Growing Conditions

Welsh onion is propagated through seeds. It has wide adaptability to its growing environment, but in order to achieve high yield and quality, suitable conditions are needed. The plants can survive heavy frosts from −45 to −25 °C. The average daily air temperature for effective growth ranges from 7 °C to 30 °C, and the most suitable daily average air temperature for growth is 13–25 °C [14,37]. The optimum temperatures for different development stages are: for seed germination, 13–20 °C; for whole plant dry matter accumulation, 19–25 °C; for leaf sheath dry matter accumulation, 13–19 °C (higher temperature at this stage will cause decreasing pseudostem and leaf quality); for vernalisation, 2–7 °C; and for reproductive growth, 15–22 °C [14]. The response of Welsh onion development to daylength is neutral. If the light is too strong, the leaves will be aged, which then will affect their edible value. As long as the crop passes through vernalisation, no matter how long the daylength is, the plant can still bolt and then flower normally. When special photoselective blue nets were used, they increased the product quality, yield and plant metabolism performance [38]. To achieve high yields and good quality, the soil needs to be well-drained and rich in organic matter, with a pH of 7.0–7.4. Welsh onion has the same requirements for mineral elements as most leafy vegetables and is very sensitive to nitrogen nutrition. A proper fertilizing with nitrogen, phosphorus and potassium is beneficial to increase the yield and fertilizer use efficiency of the Welsh onion. Among them, nitrogen has the greatest effect on the yield, followed by potassium and phosphorus [39]. The interaction of nitrogen and sulphur is beneficial to promote the dry matter accumulation and improve the quality of Welsh onion. Among them, nitrogen has the biggest effect and sulphur is beneficial to the accumulation of flavour substances [40,41,42,43]. The use of DNA markers can help to identify and measure the diversity amongst the Welsh onion cultivars [44,45]. A de novo transcriptome sequencing of the Welsh onion was performed in the study by Sun’s team, reported in 2016. Almost all known genes referring to sulphur and selenium metabolism were determined in their study, which offered an important genomic resource for future research on the Welsh onion [46]. The effects of nitrogen on the Welsh onion nitrogen-related genes expression and amino acid synthesis were studied by Zhao’s team. It was reported by them in 2021 that differently expressed genes (1665 genes), in response to different nitrogen concentrations, resulted in different amino acid contents in the plant [47]. On the other hand, appropriate application of calcium can promote the absorption of nitrogen, and silicon dioxide can increase the absorption of nitrogen, phosphorus and potassium. Both calcium and silicon dioxide can promote the growth of the Welsh onion and improve its yield and quality [48,49]. The crop is drought-tolerant but not waterlogging-tolerant. One- to two-day-long waterlogging will lead to the root rot and death [14,50]. The seedling development stage requires sufficient soil moisture. During the pseudostem development, soil moisture around the roots should not be less than 80% [14].

*Allium fistulosum* belongs to a green plant vernalisation type, i.e., specific seedling characteristics and sufficient accumulated time at low temperature are indispensable for the completion of its vernalisation process [51]. Only if these conditions for vernalisation are fulfilled, the plant will complete the flower bud formation and bolt. For this reason, seed production of this crop is limited to certain areas that can guarantee a temperature range in the winter between 3–10 °C, which includes countries located in the temperate zone. The following countries meet such growing conditions and have good seed production tradition: Italy, France, Turkey, the US, Chile, Argentina, Australia, South Africa, Russia and China [9,17,52].

## 6. Practical Use

In Asian countries, the Welsh onion is used in many ways. It could be eaten fresh or cooked. The most popular ways of eating are: stir-fried with meat, as a dish (the most common way in China); directly dipped in sauce and then eaten fresh (mainly in the northern provinces of China, such as Shandong and the three Manchurian provinces); shredded and used as an ingredient to enhance the flavour of other dishes; cut into small pieces for salads (the last two ways are widely used in many Asian dishes).

Forty different Welsh onion cultivars were selected in China for nutritional analysis in 2008. Divided by tillering habits, common Welsh onions had small crude fibre and high free amino acid contents. They were suitable for both fresh and cooked eating. Tillering Welsh onions were high in sugar, rich in protein, vitamin C, and low in pyruvic acid; they were suitable for fresh eating. Among the cultivars, drumstick-type Welsh onions had high sugar and pyruvic acid contents; they showed good flavour for both fresh and cooked eating. Japanese Welsh onions had a lower sugar content and a higher pyruvic acid content; they were suitable for eating cooked. There were also big differences reported in nutritional value amongst the different cultivars of the same type, e.g., within the tillering type, the sugar content (9.89–10.74%) and pyruvic acid content (10.70–15.05%) [53].

The Welsh onion is also used in traditional Chinese medicine thanks to its natural chemical compounds which benefit human health. Bulbs, pseudostem juice, leaves, flowers, seeds and roots have medicinal value [54], as antibacterial, antitumour, antihypertension, antiobesity, antioxidation, activation of cardiovascular system, antiplatelet aggregation, intestinal spasm relief and immune function regulation [55,56,57,58,59]. Its main active compounds include volatile oil (mostly sulphide), oleic acid, linoleic acid, allicin, pectin and vitamin C [4,57,58,59,60,61,62,63]. It was reported that the extracts of Welsh onion stalks, leaves and roots all had antioxidant effects, and the extract of stalks showed the strongest antioxidant effect [64].

## 7. Breeding Directions

Today, as the situation on the vegetable market had changed drastically, some old practices regarding the Welsh onion as a perennial are no longer important [17]. Indeed, in Japan nowadays, the annual production of this crop for a cultivar group called “Negi” (long white pseudostem), is one of the highest (in quantity) among vegetables grown in the country, following the production of tomato, strawberry and cucumber. For this reason, plant breeding and seed production companies have focused their breeding programmes on different traits more fitting to the actual growing market and producer’s needs (Table 1). They include disease resistance, yield, late bolting, improved consumption qualities (e.g., low pungency or high sugar content), and suitability for mechanized harvesting. As a result, we have high-performing hybrid cultivars adaptable to different climatic conditions, growing areas and season, and with a higher market quality [65].

**Figure 4:**
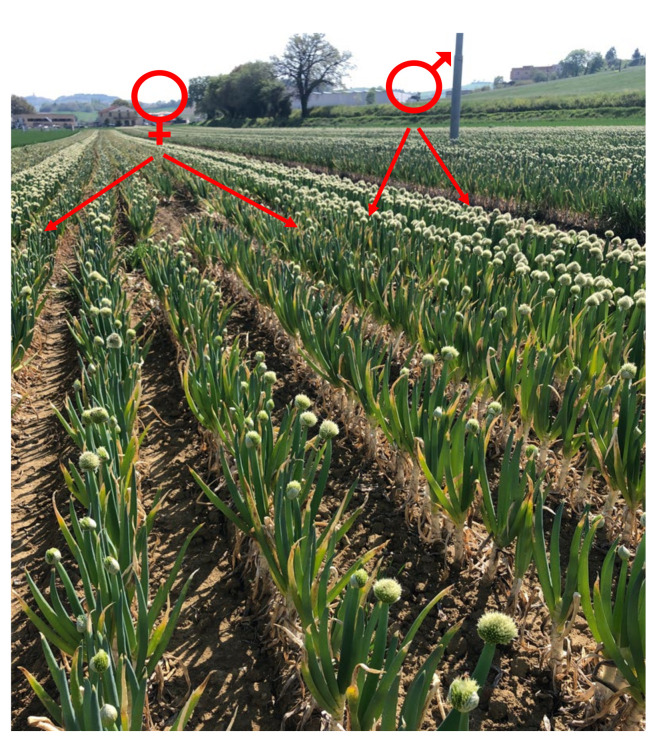
Hybrid seed production with the use of male sterile (MS) and pollinating (P) lines. The ratio of MS to P rows is 5 to 2. (Photo G. Padula).

The breeding technologies to obtain Welsh onion hybrid cultivars are similar to procedures that are used for other onion-like crops. In general, cytoplasmic male sterility (CMS) is indeed an indispensable trait in hybrid (F_1_) bunching onion breeding programmes (Figure 4). Male sterile (MS) plants were discovered from several bunching onion accessions available from the seed companies. This sterility was then transferred via intraspecific crossings in order to obtain CMS breeding materials. This, however, required the development of maintainers (complementary lines) related to the female or special A lines [66].

## 8. Commercial Production and Selection of the Proper Cultivar

Nowadays, China is the leading country in the world in Welsh onion production area with more than 500,000 ha, while South Korea and Japan each have a similar production area—around 25,000 ha. The most important seed companies developing new hybrid cultivars are all from Japan: Sakata, Takii, Kaneko, Musashino and Tokita. However, some breeding activity is also carried out in South Korea by Nongwoo Bio and other minor seed companies. In the EU and the US, the Welsh onion is not a traditional species and has been adapted for a different use, such as the spring Welsh onion, and therefore with a different commercial production technology. It provides a much higher plant density, i.e., up to 2 million plants per hectares. The market requires a product that is commonly named salad onion (or green onion), with more tender and sweeter pseudostems, long (9–11 cm) compared to what is grown in Asia, known as the “Negi” Welsh onion and characterized with pseudostem lengths from 35 to 45 cm, very similar to the leek. Germany in Europe is the country with the biggest area grown with this crop, with an estimated 1300–1400 hectares. An important part of the European fresh salad onion production though is located in North Africa, in countries such as Egypt, with a cultivation area of 4000 hectares, and Morocco with 350–400 hectares [17].

In the US and Mexico, the use of the Welsh onion is very similar to the European fresh product. Its production technology is similar to the European one, although there, it is mostly based on an interspecific hybrid between *Allium fistulosum* and *Allium cepa*.

As the crop is propagated through seeds, considering the high plant density seed cost per hectare (1200 to 1500 euros per hectare for hybrid cultivars), is an important aspect for the grower. Therefore, the cultivar selection criteria are largely defined by the objective of reducing agronomical costs (Table 2). For this reason, since harvesting of pseudostems is still carried out by hand, the easy leaf cleaning is a fundamental characteristic required by the producer. It consists in removing the first and second external leaf directly in the field, before transporting the fresh salad onion to the packing factory. Mechanical harvesting is a valid alternative to reduce this cost, but the hybrid cultivars available are not entirely adequate because of frequent leaf damage during this process and consequently high fresh product waste. The problem is still actual, until plant breeding and seed production companies are able to release new hybrid cultivars with ideal specific characteristics as mentioned in the Table 1 and Table 2.

Plant uniformity is also an important trait that allows a better field management reducing waste and consequently growing cost. Resistance against downy mildew (*Peronospora destructor* Caspary), grey leaf blight (*Botrytis squamosa* J.C. Walker) and rust (*Puccinia allii* G. Winter) are in demand by the growers, even though the use of chemicals can overcome or limit the spread of the diseases [67]. The sources of such resistance are usually found by seed companies themselves. Resistance genes might be found in gene banks or in companies’ breeding collections, but the ways to get them are not made known. Nevertheless, the new hybrid cultivars released by seed companies, and thanks also to molecular approaches that helped to clarify the genetic control of many traits, are now carrying at least one or more breeding programmes to develop disease-resistant cultivars.

When it comes to market needs, the main characters evaluated for cultivar choice are related to the leaf and pseudostem quality. Dark green leaves are well requested by consumers, while the pseudostem should be white, with a limited green part, uniform, with a diameter between 8 and 12 mm, and no bulbs formation (or very limited) on its bottom (Table 2).

## 9. Future Perspectives

In the coming years, both the cultivars quality and the production costs need to be further investigated. As the Welsh onion is propagated through seeds, the first step in the production should be to receive a higher quality sowing material. In order to achieve high cultivar quality, hybrid breeding will become a more and more important issue. Furthermore, research on more suitable and cheaper seed storage methods will ensure a high quality of Welsh onion seeds and reduce the loss of seeds caused by improper storage conditions. Disease resistance should become the second main direction of breeding. It will reduce production costs, improve product quality and moreover provide possibilities for an organic Welsh onion production. In order to further reduce production costs and allow Welsh onion products to enter the market at more competitive prices, cultivars more suitable for mechanized production and harvest should be developed.

From the market point of view, Welsh onion products will need to conform more to the consumers’ eating scene. Different traits of Welsh onions are required for their different uses, so such fitting traits should be specifically developed, e.g., higher sugar and lower pyruvic acid contents are required for fresh eating, whereas higher pyruvic acid contents are required for cooking.

As to the genetic source of the Welsh onion, one has two options: directly contacting a breeder (plant breeding and seed production companies) working with the crop in Japan, South Korea, Russia and China, or contacting a gene bank with such accessions in the same countries.

## Figures and Tables

**Table 1:** The traditional and new breeding directions of Welsh onion cultivars of main Japanese seed companies [1,2,13,16,17,51].

Traditional	New
Earliness High yield of leaves Good taste of leaves Frost resistance High vigour of plants Open-pollinated cultivars Drought resistance	Hybrid cultivars Easy leaf cleaning High yield of whole plants Good taste of whole plants Good growth in high density Long white pseudostem Violet pseudostem Resistance to downy mildew Resistance to grey mould Low ability to bolt Leaf colour (dark green) Suitable for mechanical harvest

**Table 2:** The main commercial cultivar criteria for the Welsh onion [1,2,13,16,17].

Agronomical Traits	Market Traits
Cleanability	Leaf colour
Uniformity	Shaft length
Disease resistance	Shaft diameter
Plant growth habit	No bulbs
Earliness	
